# High Prevalence of Extended-Spectrum β-Lactamase Producing Enterobacteriaceae Among Clinical Isolates From Cats and Dogs Admitted to a Veterinary Hospital in Switzerland

**DOI:** 10.3389/fvets.2018.00062

**Published:** 2018-03-27

**Authors:** Anna Lena Zogg, Sabrina Simmen, Katrin Zurfluh, Roger Stephan, Sarah N. Schmitt, Magdalena Nüesch-Inderbinen

**Affiliations:** ^1^National Centre for Enteropathogenic Bacteria and Listeria, Vetsuisse Faculty, Institute for Food Safety and Hygiene, University of Zürich, Zürich, Switzerland; ^2^Vetsuisse Faculty, Institute of Veterinary Bacteriology, University of Zürich, Zürich, Switzerland

**Keywords:** extended-spectrum β-lactamase, clinical, genotypes, cats, dogs

## Abstract

**Objectives:**

This study aimed to identify and characterize extended-spectrum β-lactamase (ESBL) producing Enterobacteriaceae among clinical samples of companion animals.

**Methods:**

A total of 346 non-duplicate Enterobacteriaceae isolates were collected between 2012 and 2016 from diseased cats (*n* = 115) and dogs (*n* = 231). The presence of *bla*_ESBL_, PMQR genes, and the azithromycin resistance gene *mph(A)* was confirmed by PCR and sequencing of *bla* genes. Isolates were further characterized by antimicrobial resistance profiling, multilocus sequence typing, phylogenetic grouping, identification of mutations in the QRDR of *gyrA* and *parC*, and screening for virulence-associated genes.

**Results:**

Among the 346 isolates, 72 (20.8%) were confirmed ESBL producers [58 *Escherichia coli (E. coli)*, 11 *Klebsiella pneumoniae (K. pneumoniae)*, and 3 *Enterobacter cloacae*]. The strains were cultured from urine (*n* = 45), skin and skin wounds (*n* = 8), abscesses (*n* = 6), surgical sites (*n* = 6), bile (*n* = 4), and other sites (*n* = 3). ESBL genes included *bla*_CTX-M-1_, _14_, _15_, _27_, _55_, and *bla*_SHV-12_, predominantly *bla*_CTX-M-15_ (54.8%, 40/73), and *bla*_CTX-M-1_ (24.7%, 18/73). Further genes included *qnrB* (4.2%, 3/72), *qnrS* (9.7%, 7/72), *aac(6*’*)-Ib-cr* (47.2%, 34/72), and *mph(A)* (38.9%, 28/72). Seventeen (23.6%) isolates belonged to the major lineages of human pathogenic *K. pneumoniae* ST11, ST15, and ST147 and *E. coli* ST131. The most prevalent ST was *E. coli* ST410 belonging to phylogenetic group C.

**Conclusion:**

The high prevalence of ESBL producing clinical Enterobacteriaceae from cats and dogs in Switzerland and the presence of highly virulent human-related *K. pneumoniae* and *E. coli* clones raises concern about transmission prevention as well as infection management and prevention in veterinary medicine.

## Introduction

Members of the family of the Enterobacteriaceae, although natural inhabitants of the intestinal tracts of mammals, may cause urinary tract, skin, ear, soft tissue, and respiratory infections in cats and dogs (1). For uncomplicated infections, first-line therapeutic options are ampicillin, amoxicillin-clavulanate or first- and second-generation cephalosporins, while amikacin, third-generation cephalosporins or fluoroquinolones (enrofloxacin or ciprofloxacin) remain appropriate for severe infections (1, 2). One of the most important mechanisms of antimicrobial resistance in Enterobacteriaceae is the enzymatic inactivation of penicillins and cephalosporins by means of plasmid-mediated extended-spectrum β-lactamases (ESBLs), such as the TEM-, SHV-, or cefotaxime (CTX)-M-group enzymes (3). The emergence of ESBL producing Enterobacteriaceae in healthy and in diseased companion animals constitutes an increasing challenge to infection management in veterinary therapy. Moreover, resistance caused by ESBLs is often associated with resistance to other classes of antibiotics like aminoglycosides, fluoroquinolones, and sulfamethoxazole/trimethoprim (SXT), which are antimicrobials that are critically important in human medicine (4, 5). Additionally, previous studies have shown that multidrug resistant, highly virulent human-related clonal lineages of Enterobacteriaceae, such as *Escherichia coli (E. coli)*, belonging to sequence type (ST)131 and ST648, or *Klebsiella pneumoniae (K. pneumoniae)* ST11, ST15, and ST147 may be isolated from companion animals (6, 7). Consequently, there is growing concern that ESBL producers in companion animals pose a potential health hazard to humans, either through direct transmission of resistant pathogens from animals to humans, or indirectly through transmission of resistance genes (8, 9). Recent data on the prevalence of ESBL producers in clinical isolates of cats and dogs and the phenotypes and genotypes of such isolates are scarce for Switzerland, and it remains unclear to what extent clinically relevant phylogenetic or clonal lineages occur.

Here, we analyze a collection of clinical feline and canine Enterobacteriaceae obtained during 2012–2016 by (i) identifying ESBL producers within the strain collection, (ii) assessing their antimicrobial resistance profiles, (iii) determining their *bla*_ESBL_ genes and screening for plasmid-mediated fluoroquinolone and azithromycin resistance genes, and by (iii) characterizing *E. coli* and *K. pneumoniae* strains by multilocus sequence typing (MLST), and *E. coli* strains by phylogenetic grouping and virulence gene profiling.

## Materials and Methods

### Bacterial Isolates

Between 2012 and 2016, 346 clinical Enterobacteriaceae were isolated from diseased cats (*n* = 115) and dogs (*n* = 231) admitted to the veterinary clinic of the University of Zürich. The isolates were cultured from urinary samples (*n* = 273), samples obtained from surgical sites (*n* = 26), abscess samples (*n* = 16), skin and skin wound samples (*n* = 14), bile samples (*n* = 7), and samples from other sites (*n* = 10). Strain identification and routine antimicrobial susceptibility profiling was performed using the VITEK^®^ two compact system with AST GN38 cards (Biomérieux, Nürtingen, Germany) according to the manufacturer’s instructions. The identity of *Enterobacter cloaca*e (*E. cloacae*) was confirmed by matrix-assisted laser desorption/ionization time-of-flight mass spectrometry (MALDI-TOF–MS, Bruker Daltronics, Bremen, Germany). ESBL producers were screened by using the chromogenic medium Brilliance™ ESBL Agar (Oxoid, Hampshire, UK), according to the manufacturer’s recommendations. All non-duplicate isolates growing on ESBL agar were further analyzed. In accordance with local legislation, ethics approval was not required for this study.

### Identification of *bla*_ESBL_ Genes and Antibiotic Susceptibility Testing

The presence of *bla*_ESBL_ genes was established by PCR, and amplicons were sequenced as described previously using primers listed in Table S1 in Supplementary Material (10–12). For the detection of the CTX-M-25 enzyme group, the newly designed primers Gr. 25 CTX-M fw CCTGTGTTTCGCTGCTGTTGG and Gr. 25 CTX-M rv GGCTCTCTGCCTTCGGCTCC, were used.

Antimicrobial susceptibility testing was performed according to Clinical and Laboratory Standards Institute (CLSI) performance standards (13), using the disk-diffusion method and the antibiotics ampicillin (AM), amoxicillin with clavulanic acid (AMC), azithromycin (AZM), cefazolin, cefepime, CTX, chloramphenicol (C), ciprofloxacin (CIP), fosfomycin (FOS), gentamicin (G), kanamycin (K), nalidixic acid (NA), nitrofurantoin (F/M), streptomycin (S), SXT, and tetracycline (TE) (Becton Dickinson, Allschwil, Switzerland). Results were interpreted according to CLSI standards (13). For azithromycin, an inhibition zone of ≤12 mm was interpreted as resistant. Isolates displaying resistance to three or more classes of antimicrobials (counting β-lactams as one class) were defined as multidrug-resistant (MDR).

### Identification of Additional Antimicrobial Resistance Genes

The plasmid-mediated fluoroquinolone resistance genes *aac(6*'*)-Ib-cr, qnrA, qnrB, qnrC, qnrD, qnrS*, and *qepA*, and the plasmid-mediated azithromycin resistance gene *mph(A)* were detected by PCR as described elsewhere using primers listed in Table S1 in Supplementary Material (14, 15).

Quinolone-resistant *E. coli* strains were examined for mutations in the quinolone resistance-determining regions (QRDRs) of *gyrA* and *parC*, using PCR amplification and sequencing primers as described previously using primers listed in Table S1 in Supplementary Material (14).

Synthesis of primers and DNA custom sequencing was carried out by Microsynth (Balgach, Switzerland) and nucleotide sequences were analyzed with CLC Main Workbench 6.6.1. For database searches, the BLASTN program of NCBI^1^ was used.

### Phylogenetic Characterization and MLST

Phylogenetic classification of the *E. coli* isolates into one of the eight groups, including A, B1, B2, C, D, E, F, (*E. coli sensu stricto)*, or *Escherichia* clade I, was performed as described by Clermont et al. (16).

Sequence type determination of the *E. coli* isolates was carried out as described by Wirth et al. (17). Sequences were imported into the *E. coli* MLST database website^2^ to determine MLST types. Alleles and STs that had not been previously described were termed new ST, but not assigned new numerical designations by the database.

Sequence type determination of the *K. pneumoniae* isolates was performed according to previously described methods (18). STs were determined according to the *Klebsiella* MLST database.^3^

### Virulence Factor (VF) Determination in Uropathogenic *E. coli* Isolates

*Escherichia coli* isolated from urinary samples were tested by conventional PCR for the presence of virulence-associated genes that mediate adhesion (*p*-fimbrial adhesion genes *papAH* and *papEF*, and the chaperone-usher fimbria *yfcv*), toxins (α-hemolysin *hlyA*), siderophores (the ferric yersiniabactin uptake protein *fyuA*), serum resistance (*traT*), and the right-hand terminus of pathogenicity island (PAI) from *E. coli* strain CFT073, using primers listed in Table S1 in Supplementary Material and conditions described previously (19, 20). The aggregate VF score was defined as the number of unique VF detected for each isolate, counting the PAI marker as one.

## Results

During 2012–2016, 20.8% (72/346) of clinical Enterobacteriaceae isolated from cats and dogs were ESBL producers. The isolates originated from 7 cats and 65 dogs, amounting to 6% (7/115) of the feline and 28.1% (65/231) of the canine isolates, respectively. The prevalence of ESBL producers was remarkably higher among isolates from dogs than from cats. Overall, ESBL producers (58 *E. coli*, 11 *K. pneumoniae*, and 3 *E. cloacae*) were cultured from 16.5% (45/273) of the urinary samples, 57.1% (8/14) of the skin and skin wound samples, 37.5% (6/16) of abscess samples, 23% (6/26) of the samples obtained from surgical sites, 57.1% (4/7) of bile samples, and 30% (3/10) of the samples from other sites (Table 1). Among the *E. coli* from urinary samples, 17% (35/205) were ESBL producers (Table 1).

**Table 1:** Percent and distribution of extended-spectrum β-lactamases (ESBL) producers among clinical Enterobacteriaceae from cats and dogs in Switzerland, 2012–2016.

**Host**	**Source**	*Escherichia coli*			*Klebsiella pneumoniae*			*Enterobacter cloacae*			Other species^a^			Total Enterobacteriaceae		
						
		No.	No. ESBL	% ESBL	No.	No. ESBL	% ESBL	No.	No. ESBL	% ESBL	No.	No. ESBL	% ESBL	No.	No. ESBL	% ESBL
Cats	Urine	74	1	1.4	6	2	33.3	10	0	0	6	0	0	96	3	3.1
Cats	Surgical sites	4	1	25	0	0	0	2	0	0	0	0	0	6	1	16.6
Cats	Abscess	3	1	33.3	0	0	0	2	0	0	0	0	0	5	1	20
Cats	Wound/skin	3	1	33.3	0	0	0	0	0	0	0	0	0	3	1	33.3
Cats	Bile	3	0	0	0	0	0	0	0	0	0	0	0	3	0	0
Cats	Other	2	1	50	0	0	0	0	0	0	0	0	0	2	1	50
Dogs	Urine	131	34	26	25	5	20	10	3	30	11	0	0	177	42	23.7
Dogs	Surgical sites	13	5	38.5	3	0	0	4	0	0	0	0	0	20	5	25
Dogs	Abscess	6	4	66.7	3	1	33.3	0	0	0	2	0	0	11	5	45.5
Dogs	Wound/skin	6	5	83.3	2	2	100	1	0	0	2	0	0	11	7	63.6
Dogs	Bile	4	4	100	0	0	0	0	0	0	0	0	0	4	4	100
Dogs	Other	5	1	20	1	1	100	2	0	0	0	0	0	8	2	25

*^a^Other species included Citrobacter freundii, Citrobacter koseri, Proteus mirabilis, and Proteus vulgaris*.

In addition to their resistance to penicillins and extended-spectrum cephalosporins, the isolates were frequently resistant to quinolones and fluoroquinolones, with 88.9% (64/72) resistant to NA and 83.3% (60/72) resistant to ciprofloxacin. They were also resistant to SXT (76.4%, 55/72), TE (72.2%, 52/72), aminoglycosides streptomycin (45.8%, 33/72), gentamycin (37.5%, 27/72), kanamycin (19.4%, 14/72), chloramphenicol (25%, 18/72), as well as to azithromycin (22.2%, 16/72), and to nitrofurantoin (12.5%, 9/72). One *K. pneumoniae* isolate (1.4%) was resistant to fosfomycin. Overall, 73.6% (53/72) were MDR and none was pansusceptible (Table S2 in Supplementary Material).

In total, 73 ESBL genes were detected among the 72 isolates, including in 1 *K. pneumoniae* isolate co-harboring *bla*_CTX-M-15_ and *bla*_SHV-12_ (Table 2). Among the ESBL genes, *bla*_CTX-M-15_ predominated (54.8%, 40/73), followed by *bla*_CTX-M-1_ (24.7%, 18/73). Other ESBL genes included *bla*_CTX-M-55_ (6.8%, 5/73), *bla*_CTX-M-14_ and *bla*_SHV-12_ (each 5.5%, 4/73), and *bla*_CTX-M-27_ (2.7%, 2/73).

**Table 2:** Type and distribution of extended-spectrum β-lactamases (ESBL) genes and other plasmid-mediated resistance genes among 72 clinical Enterobacteriaceae isolated from cats and dogs in Switzerland, 2012–2016.

Host	Species	No. of isolates	Source (*n*)	MLST (*n*)	*bla*_ESBL_	Additional plasmid-mediated AMR determinants
Cat	*Escherichia coli (E. coli)*	1	Urine (1)	10 (1)	*bla*_CTX-M-1_	−
Cat	*E. coli*	1	Other (1)	23 (1)	*bla*_CTX-M-1_	*mph(A), qnrS*
Cats	*E. coli*	3	Abscess (1), wound (1), surgical site (1)	361 (2), 648 (1)	*bla*_CTX-M-15_	*mph(A), aac(6*'*)-Ib-cr*
Cat	*Klebsiella pneumoniae (K. pneumoniae)*	1	Urine (1)	15 (1)	*bla*_CTX-M-15_	*aac(6*'*)-Ib-cr*
Cat	*K. pneumoniae*	1	Urine (1)	147 (1)	*bla*_CTX-M-15_	*qnrS*
Dogs	*E. coli*	13	Urine (9), abscess (1), wound (1), surgical site (2)	58 (1),101 (2), 117 (1), 410 (5), 617 (2), 1431 (1), new ST (1)	*bla*_CTX-M-1_	−
Dog	*E. coli*	1	Surgical site (1)	3,889 (1)	*bla*_CTX-M-1_	*mph(A)*,
Dog	*E. coli*	1	Bile (1)	90 (1)	*bla*_CTX-M-1_	*mph(A), aac(6*'*)-Ib-cr*
Dog	*K. pneumoniae*	1	Urine (1)	788 (1)	*bla*_CTX-M-1_	*mph(A), qnrB*
Dogs	*E. coli*	2	Urine (2)	744 (2)	*bla*_CTX-M-14_	−
Dogs	*E. coli*	2	Urine (1), wound (1)	744 (1), 131 (1)	*bla*_CTX-M-14_	*mph(A)*
Dogs	*E. coli*	3	Urine (3)	131 (1), 354 (1), 648 (1)	*bla*_CTX-M-15_	−
Dog	*E. coli*	1	Urine (1)	533 (1)	*bla*_CTX-M-15_	*mph(A)*
Dogs	*E. coli*	10	Urine (7), bile (1), wound (1), surgical site (1)	131 (3), 410 (7)	*bla*_CTX-M-15_	*aac(6*'*)-Ib-cr*
Dogs	*E. coli*	12	Urine (5), abscess (3), bile (1), wound (1), surgical site (1), other (1)	131 (1), 167 (2), 361 (6), 410 (2), new ST (1)	*bla*_CTX-M-15_	*mph(A), aac(6*'*)-Ib-cr*
Dog	*E. coli*	1	Urine (1)	New ST (1)	*bla*_CTX-M-15_	*mph(A), aac(6*'*)-Ib-cr, qnrB*
Dogs	*K. pneumoniae*	4	Urine (3), other (1)	147 (4)	*bla*_CTX-M-15_	*qnrS*
Dogs	*K. pneumoniae*	2	Wound (2)	15 (2)	*bla*_CTX-M-15_	*aac(6*'*)-Ib-cr*
Dog	*K. pneumoniae*	1	Abscess (1)	11 (1)	*bla*_CTX-M-15_	*mph(A), aac(6*'*)-Ib-cr, qnrB*
Dog	*K. pneumoniae*	1	Urine (1)	147 (1)	*bla*_CTX-M-15_, *bla*_SHV-12_	*qnrS*
Dog	*E. coli*	1	Urine (1)	131 (1)	*bla*_CTX-M-27_	*mph(A)*
Dog	*E. coli*	1	Bile (1)	648 (1)	*bla*_CTX-M-27_	*mph(A), aac(6*'*)-Ib-cr*
Dogs	*E. coli*	3	Urine (2), wound (1)	457 (2), 1177 (1)	*bla*_CTX-M-55_	−
Dogs	*E. coli*	2	Urine (2)	410 (2)	*bla*_CTX-M-55_	*mph(A), aac(6*'*)-Ib-cr*
Dogs	*Enterobacter cloacae*	3	Urine (3)	–	*bla*_SHV-12_	*qnrA*

*aac(6’)-Ib-cr, aminoglycoside 6′-N-acetyltransferase variant; AMR, antimicrobial resistance; bla, β-lactamase gene; MLST, multilocus sequence typing; mph(A), macrolide 2′-phosphotransferase gene; qnr, quinolone resistance gene; –, not determined; −, not present*.

In addition to *bla*_ESBLs_, other plasmid-mediated resistance genes detected among the 72 isolates included *aac(6*’*)-Ib*-cr (47.2%, 34/72), *mph(A)* (38.9%, 28/72), *qnrS* (9.7%, 7/72), *qnrA* and *qnrB* (each 4.2%, 3/72) (Table 2).

The majority of the *aac(6*’*)-Ib-cr* genes (88.2%, 30/34), the *mph(A)* genes (62%, 18/29), the *qnrB* (66.7%, 2/3), and *qnrS* genes (85.7%, 6/7) was detected in isolates harboring *bla*_CTX-M-15_. All *qnrA* were detected together with *bla*_SHV-12_ in *E. cloacae* (Table 2).

Phylogenetic analysis of the 58 *E. coli* isolates revealed a predominance of group C (32.8%, 19/58), followed by group A (31%, 18/58), group B2 and group F (each 12%, 7/58), group B1 (8.6%, 5/58), and group D (3.4%, 2/58) (Table S2 in Supplementary Material).

Among the 58 *E. coli* isolates, 23 different STs and three new STs were identified (Table 2; Table S3 in Supplementary Material). Most frequently, isolates belonged to ST410 (27.6%, 16/58), followed by a collective of STs occurring only once or twice (24.1%, 14/58), ST361 (13.8%, 8/58), ST131 (12%, 7/58), and ST648, ST744, and new STs (each 5.2%, 3/58). *E. coli* ST410 and human-related pandemic clone *E. coli* ST131 were detected only in isolates from dogs. *E. coli* ST410 was isolated from 33.3% of the urine samples from dogs.

Among the 11 *K. pneumoniae* isolates, 4 different STs were detected (Table 2; Table S2 in Supplementary Material). The majority (54.5%, 6/11) of the isolates belonged to ST147. Other STs included ST15 (27.3%, 3/11), ST11, and ST788 (both 9.1%, 1/11).

Among the 51 *E. coli* isolates displaying quinolone resistance, all revealed chromosomal mutations that result in amino acid substitutions in GyrA and ParC. Unusual point mutations Asp87→Tyr in GyrA and Glu84→Gly in ParC were noted for two *E. coli* ST457 isolates harboring *bla*_CTX-M-55_ (Table 3; Table S2 in Supplementary Material).

**Table 3:** Amino acid substitutions in the QRDR of 51 quinolone-resistant extended-spectrum β-lactamases producing *Escherichia coli* from cats and dogs in Switzerland, 2012–2016.

Host (*n* = 51)	QRDR					
	
	*gyrA*			*parC*		
		
Ser83→Leu *n* (%)	Asp87→Asn *n* (%)	Asp87→Tyr *n* (%)	Ser80→Ile *n* (%)	Glu84→Val *n* (%)	Glu84→Gly *n* (%)
Cats (*n* = 4)	4 (100)	3 (75)	0 (0)	0 (75)	0 (0)	0 (0)
Dogs (*n* = 47)	47 (100)	44 (93.6)	2 (4.3)	46 (97.9)	7 (14.9)	1 (2.1)

*Asn, asparagine; Asp, aspartic acid; CIP, ciprofloxacin; Glu, glutamic acid; Gly, glycine; *gyrA*, DNA gyrase (type II topoisomerase) gene; Ile, isoleucine; Leu, leucine; *parC*, topoisomerase IV gene; QRDR, quinolone resistance determining region; Ser, serine; Tyr, tyrosine; Val, valine*.

Virulence factors were distributed unequally among the 35 uropathogenic *E. coli* isolates (Table 4).

**Table 4:** Virulence-associated genes detected in 35 uropathogenic extended-spectrum β-lactamases producing *Escherichia coli* from cats and dogs in Switzerland, 2012–2016.

Host	No. of isolates	PG	ST	CC	*papAH*	*papEF*	*yfcv*	*hlyA*	*fyuA*	*traT*	PAI	Plasmid-mediated resistance gene(s)
Dog	1	A	617	10	+	+	−	+	+	+	+	*bla*_CTX-M-1_
Dog	1	A	617	10	+	+	−	−	+	+	+	*bla*_CTX-M-1_
Dog	2	A	361	–	−	−	−	−	−	−	−	*aac(6*'*)-Ib-cr, bla*_CTX-M-15_, *mph(A)*
Dog	1	A	361	–	−	−	−	−	+	+	−	*aac(6*’*)-Ib-cr, bla*_CTX-M-15_, *mph(A)*
Dog	1	A	744	–	–	−	−	−	−	+	−	*mph(A), bla*_CTX-M-14_
Dog	2	A	744	–	−	−	−	−	−	−	−	*bla*_CTX-M-14_
Dog	1	B1	533	–	−	−	−	−	−	+	−	*mph(A), bla*_CTX-M-15_
Dog	1	B1	1431	–	−	−	−	+	+	+	−	*bla*_CTX-M-1_
Dog	2	B2	131	131	+	+	+	+	+	+	+	*aac(6*’*)-Ib-cr, bla*_CTX-M-15_
Dog	1	B2	131	131	+	+	+	+	+	+	+	*bla*_CTX-M 15_
Dog	1	B2	131	131	−	−	+	−	+	+	+	*mph(A), bla*_CTX-M-27_
Cat	1	C	23	23	−	−	−	−	+	−	−	*bla*_CTX-M-1_
Dog	5	C	410	23	−	−	−	−	−	−	−	*bla*_CTX-M-1_
Dog	5	C	410	23	−	−	−	−	−	−	−	*aac(6*’*)-Ib-cr, bla*_CTX-M-15_
Dog	1	C	410	23	+	−	−	−	+	−	−	*aac(6*’*)-Ib-cr, bla*_CTX-M-15_, *mph(A)*
Dog	1	C	410	23	−	−	−	−	−	−	−	*aac(6*’*)-Ib-cr, bla*_CTX-M-15_, *mph(A)*
Dog	2	C	410	23	−	−	−	+	−	−	−	*aac(6*’*)-Ib-cr, bla*_CTX-M-55_, *mph(A)*
Dog	1	C	nd	nd	−	−	−	+	−	−	−	*aac(6*’*)-Ib-cr, bla*_CTX-M-15_, *mph(A), qnrB*
Dog	1	D	1177	–	−	−	−	−	+	+	−	*bla*_CTX-M-55_
Dog	1	F	117	–	−	−	−	−	−	+	−	*bla*_CTX-M-1_
Dog	1	F	354	354	−	−	+	−	−	+	−	*bla*_CTX-M-15_
Dog	1	F	457	–	−	−	+	−	−	+	+	*bla*_CTX-M-55_
Dog	1	F	648	648	−	−	+	−	+	+	+	*bla*_CTX-M-15_

*aac(6’)-Ib-cr, aminoglycoside 6'-N-acetyltransferase variant; bla, β-lactamase gene; CC, clonal complex; fyuA, ferric yersiniabactin uptake protein gene; hlyA, α-hemolysin gene; mph(A), macrolide 2′-phosphotransferase gene; nd, not determined; PAI, right-hand terminus of pathogenicity island; papAH and papEF, p-fimbrial adhesion genes; PG, phylogenetic group; qnrB, quinolone resistance gene; ST, sequence type; traT, serum resistance gene; yfcv, chaperone-usher fimbria gene; +, presence of a trait; −, absence of a trait; –, not applicable*.

For 42.9% (15/35) of *E. coli* urinary isolates, no VF was detected. Strains with aggregate VF score ≥1 were identified in 34.5% (57.1%/35) of the isolates. VF scores were highest for isolates belonging to ST617 (median 5.5, range 5–6) and ST131 (median 7, range 4–7).

## Discussion

This study identified a high prevalence (20.8%) of ESBL-producing Enterobacteriaceae derived from clinical samples of cats and dogs collected during 2012–2016 at the veterinary clinic of the University of Zürich, Switzerland. This is considerably higher than that found in similar studies from pets in the UK (7%) (21), the Netherlands (2%) (22), and France (3.7%) (23), and remarkably higher than the prevalence of 1.6% detected in a European collection of Enterobacteriaceae obtained from diseased companion animals in 2015 (6). In addition, among the uropathogenic *E. coli* analyzed in this study, the observed prevalence of 16.8% of ESBL producers is considerably higher than that found previously in cats and dogs in Switzerland between 2010 and 2012 (7.5%) (24). Although our data are single-institution based and thus limited, they provide important information on the trends in the burden of infections due to ESBL producers in veterinary medicine in Switzerland.

Overall, a diversity of *bla*_ESBL_ genes was found within three bacterial species. The predominance of *bla*_CTX-M-15_, which is highly prevalent in ESBL producers in humans, is comparable to what is found in other studies on isolates from companion animals (21, 23, 25). This gene was the only one that was detected in cats and dogs in Switzerland between 2010 and 2012 (24). Our study shows that in the following years, *bla*_CTX-M-1_, *bla*_CTX-M-14_, *bla*_CTX-M-27_, *bla*_CTX-M-55_, and *bla*_SHV-12_ harboring Enterobacteriaceae have emerged in cats and dogs in Switzerland.

Second to *bla*_CTX-M-15_, *bla*_CTX-M-1_ was the most frequent variant identified in this study. The *bla*_CTX-M-1_ gene is the most prevalent *bla*_ESBL_ gene among ESBL-producing Enterobacteriaceae isolated food-producing animals and food, in particular chicken and chicken meat (26, 27). Consumption of raw meat represents a risk factor for dogs acquiring pathogenic *E. coli*, including ESBL producers (28, 29). Moreover, a recent study detected a high prevalence (77.8%) of ESBL producers in raw cat food and demonstrated a strong association of consumption of raw cat food with shedding of ESBL producers by household cats in the Netherlands (30). Further studies are needed to investigate the possibility of raw meat as an origin of the high prevalence of ESBL and the occurrence of CTX-M-1 producers in isolates from companion animals in Switzerland. Similarly, CTX-M-55 has been widely reported in food-producing animals and pets in mainland China (31). This ESBL variant has rarely been detected outside China and its emergence in pets in Switzerland, possibly due to international food and animal trade, warrants attention.

This study identified 17 (23.6%) isolates belonging to major lineages of human pathogenic *K. pneumoniae* and *E. coli*. CTX-M-15 producing *K. pneumoniae* ST11, ST 15, and ST147 represent major international high-risk nosocomial clones (32). *K. pneumoniae* ST11 and ST15 from companion animals have been involved in nosocomial events in veterinary clinics (7, 33). By contrast, *K. pneumoniae* ST147 has only very recently been detected in pets in Europe and in Japan (34, 35), and this is to our knowledge the second report on this ST isolated from dogs in Europe.

Pandemic human pathogenic *E. coli* ST131-producing CTX-M-15 has disseminated globally in hospital and community settings causing a wide spectrum of infections, including urinary tract infection, cystitis, pyelonephritis, and bacteremia, with transmission between humans and their companion animals (cats and dogs in particular) was well documented (36). Since the earlier study period 2010–2012 (24), the prevalence of ESBL-producing uropathogenic *E. coli* ST131 among feline and canine samples in Switzerland has increased from 0 to 1.5% (4/273), and includes *E. coli* ST131-CTX-M-15 as well as ST131-CTX-M-27, which is currently emerging in human medicine in Germany, France, and Japan (37, 38).

Other human-related strains detected in this study included *E. cloacae* harboring *bla*_SHV-12_ together with the plasmid-mediated quinolone resistance gene *qnrA*. The combined presence of *bla*_SHV-12_ and *qnrA* has been described in human clinical *E. cloacae* isolates in hospitals in France and the UK (39, 40). Although data on ESBL-producing *E. cloacae* in animals are scarce (22, 41), our results provide evidence that this important pathogen has emerged in companion animals in Switzerland, illustrating their potential for increased dissemination.

In this study, the identification of phylogenetic groups among the *E. coli* isolates was performed based on the new Clermont scheme (16). Consequently, a number of STs from this study were classified as phylogenetic group F from their original D designation, including *E. coli* ST117 which is a recognized avian pathogenic lineage (42), *E. coli* ST354 and ST648, which are frequently detected in humans and animals (9, 43), and the rarely described *E. coli* ST457. In this study, we detected two isolates belonging to ST457, both harboring the uncommon *bla*_CTX-M-55_. *E. coli* ST457-CTX-M-55 harboring the carbapenemase gene *bla*_KPC-3_ was isolated in Italy from a human diagnosed with pneumonia (44), but to our knowledge, this ST has not been associated with disease in companion animals before.

A large number (26.4%, 19/72) of isolates changed designation from the original phylogenetic group A to group C. Most isolates in this group belonged to ST410 and were of low virulence. However, the panel of VFs selected for this study was limited in number and represents only a subset of known VFs. Other important determinants of virulence may have been missed due to this limitation. Nevertheless, the pathogenic potential of ST410 has been documented previously, together with strong evidence for clonal dissemination of *E. coli* ST410 between the avian wildlife, humans, and companion animals in Germany (45, 46). CTX-M-15-producing *E. coli* ST410 was also identified as a veterinary hospital strain in the UK (21). Although currently available reports on *bla*_ESBLs_ in ST410 are limited to *bla*_CTX-M-15_, our results demonstrate that this ST can also harbor *bla*_CTX-M-1_ and *bla*_CTX-M-55_, both variants that occur among food-producing animals (26, 31). Here, we provide further evidence for the pathogenic potential of this ST in companion animals and suggest that, in addition to its potential as an international clone for the dissemination of *bla*_CTX-M-15_, it may contribute to the dispersion of other resistance genes, including other *bla*_ESBL_ variants, *aac(6*’*)-Ib-cr*, and *mph(A)*. The high prevalence (38.9%) of isolates harboring plasmid-mediated *mph(A)* which confers reduced susceptibility to azithromycin is of concern, since this macrolide is considered a last-resort antimicrobial agent for shigellosis (47). Furthermore, azithromycin represents an option for the treatment of Gram-negative rods expressing MDR, including carbapenem-resistant isolates of *Pseudomonas aeruginosa, K. pneumoniae*, and *Acinetobacter baumannii* (48), and is the only antimicrobial under consideration for the treatment of enterohemorrhagic *E. coli* in humans (49).

In conclusion, this study provides information on the prevalence, the *bla*_ESBL_ variants and the genotypes of ESBL-producing isolates in cats and dogs in Switzerland. The occurrence of potentially high-risk human-related *K. pneumoniae* and *E. coli* clones, as well as *E. cloacae* harboring *bla*_SHV-12_ and *qnrA* genes, previously described in humans suggests transmission events between companion animals as well as the possibility of the presence of a common source. This collection of ESBL-producing Enterobacteriaceae from cats and dogs identifies *E. coli* phylogroup C ST410 as a frequent MDR, ESBL-producing clone among clinical isolates from dogs in Switzerland that warrants further attention. The clinical significance of phylogroup C strains as etiological agents of extraintestinal disease and disseminators of antimicrobial resistance in companion animals remains to be investigated. Understanding the epidemiological and molecular features of ESBL-producing Enterobacteriaceae in companion animals can be helpful for infection management and prevention in veterinary as well as in human medicine.

## Author Contributions

RS designed the study. AZ, KZ, SNS, and SS carried out the microbiological and molecular biological tests. AZ, KZ, SNS, and MN-I analyzed and interpreted the data. MN-I drafted the manuscript. All authors read and approved the final manuscript.

## Conflict of Interest Statement

The authors declare that the research was conducted in the absence of any commercial or financial relationships that could be construed as a potential conflict of interest.

## Footnotes

^1^http://www.ncbi.nlm.nih.gov/blast/ (Accessed: May 17, 2017).

^2^http://enterobase.warwick.ac.uk (Accessed: June 6, 2017).

^3^http://bigsdb.pasteur.fr/klebsiella/ (Accessed: June 24, 2017).

## References

[1] Koenig A Gram-negative bacterial infection. In: Greene CE, editor. Infectious Diseases of the Dog and Cat. St Louis: Elsevier Saunders (2012). p. 349–59.

[2] Weese JS Blondeau JM Boothe D Breitschwerdt EB Guardabassi L Hillier A Antimicrobial use guidelines for treatment of urinary tract disease in dogs and cats: antimicrobial guidelines working group of the international society for companion animal infectious diseases. Vet Med Int (2011) 2011:263768 10.4061/2011/263768 21776346 PMC3134992

[3] Bush K Proliferation and significance of clinically relevant β-lactamases. Ann N Y Acad Sci (2013) 1277:84–90. 10.1111/nyas.12023 23346859

[4] Coque TM Novais A Carattoli A Poirel L Pitout J Peixe L Dissemination of clonally related *Escherichia coli* strains expressing extended-spectrum beta-lactamase CTX-M-15. Emerg Infect Dis (2008) 14:195–200. 10.3201/eid1402.070350 18258110 PMC2600198

[5] World Health Organization. Critically Important Antimicrobials for Human Medicine – 5th Revision, 2016. Geneva: World Health Organization (2017). Available from: http://apps.who.int/iris/bitstream/10665/255027/1/9789241512220-eng.pdf?ua=1 (Accessed: February 01, 2018).

[6] Bogaerts P Huang TD Bouchahrouf W Bauraing C Berhin C El Garch F Characterization of ESBL- and AmpC-producing Enterobacteriaceae from diseased companion animals in Europe. Microb Drug Resist (2015) 21:643–50. 10.1089/mdr.2014.0284 26098354

[7] Ewers C Stamm I Pfeifer Y Wieler LH Kopp PA Schønning K Clonal spread of highly successful ST15-CTX-M-15 *Klebsiella pneumoniae* in companion animals and horses. J Antimicrob Chemother (2014) 69:2676–80. 10.1093/jac/dku217 24974381

[8] Pomba C Rantala M Greko C Baptiste KE Catry B van Duijkeren E Public health risk of antimicrobial resistance transfer from companion animals. J Antimicrob Chemother (2017) 72:957–68. 10.1093/jac/dkw481 27999066

[9] Ewers C Bethe A Stamm I Grobbel M Kopp PA Guerra B CTX-M-15-D-ST648 *Escherichia coli* from companion animals and horses: another pandemic clone combining multiresistance and extraintestinal virulence? J Antimicrob Chemother (2014) 69:1224–30. 10.1093/jac/dkt516 24398338

[10] Geser N Stephan R Korczak BM Beutin L Hächler H. Molecular identification of extended-spectrum-β-lactamase genes from Enterobacteriaceae isolated from healthy human carriers in Switzerland. Antimicrob Agents Chemother (2012) 56:1609–12. 10.1128/AAC.05539-11 22155836 PMC3294945

[11] Woodford N Fagan EJ Ellington MJ Multiplex PCR for rapid detection of genes encoding CTX-M extended-spectrum β-lactamases. J. Antimicrob Chemother (2006) 57:154–5. 10.1093/jac/dki412 16284100

[12] Zurfluh K Nüesch-Inderbinen M Morach M Berner AZ Hächler H Stephan R Extended-spectrum ß-lactamase-producing-Enterobacteriaceae in vegetables imported from the Dominican Republic, India, Thailand and Vietnam. Appl Environ Microbiol (2015) 81:3115–20. 10.1128/AEM.00258-15 25724954 PMC4393435

[13] Clinical and Laboratory Standards Institute. Performance Standards for Antimicrobial Susceptibility Testing. 26th ed Wayne, PA: Clinical and Laboratory Standards Institute (2016). CLSI SupplementM100S.

[14] Zurfluh K Abgottspon H Hächler H Nüesch-Inderbinen M Stephan R Quinolone resistance mechanisms among extended-spectrum beta-lactamase (ESBL) producing *Escherichia coli* isolated from rivers and lakes in Switzerland. PLoS One (2014) 9(4):e95864 10.1371/journal.pone.0095864 24755830 PMC3995870

[15] Ojo KK Ulep C Van Kirk N Luis H Bernardo M Leitao J The mef(A) gene predominates among seven macrolide resistance genes identified in gram-negative strains representing 13 genera, isolated from healthy Portuguese children. Antimicrob Agents Chemother (2004) 48:3451–6. 10.1128/AAC.48.9.3451-3456.2004 15328110 PMC514787

[16] Clermont O Christenson JK Denamur E Gordon DM. The Clermont *Escherichia coli* phylo-typing method revisited: improvement of specificity and detection of new phylo-groups. Environ Microbiol Rep (2013) 5:58–65. 10.1111/1758-2229.12019 23757131

[17] Wirth T Falush D Lan R Colles F Mensa P Wieler LH Sex and virulence in *Escherichia coli*: an evolutionary perspective. Mol Microbiol (2006) 60:1136–51. 10.1111/j.1365-2958.2006.05172.x 16689791 PMC1557465

[18] Diancourt L Passet V Verhoef J Grimont PAD Brisse S. Multilocus sequence typing of *Klebsiella pneumoniae* nosocomial isolates. J Clin Microbiol (2005) 43:4178–82. 10.1128/JCM.43.8.4178-4182.2005 16081970 PMC1233940

[19] Johnson JR Stell AL. Extended virulence genotypes of *Escherichia coli* strains from patients with urosepsis in relation to phylogeny and host compromise. J Infect Dis (2000) 181:261–72. 10.1086/315217 10608775

[20] Spurbeck RR Dinh PC Walk ST Stapleton AE Hooton TM Nolan LK *Escherichia coli* isolates that carry *vat, fyuA, chuA*, and *yfcV* efficiently colonize the urinary tract. Infect Immun (2012) 80:4115–22. 10.1128/IAI.00752-12 22966046 PMC3497434

[21] Timofte D Maciuca IE Williams NJ Wattret A Schmidt V. Veterinary hospital dissemination of CTX-M-15 extended-spectrum beta-lactamase-producing *Escherichia coli* ST410 in the United Kingdom. Microb Drug Resist (2016) 22:609–15. 10.1089/mdr.2016.0036 27314838 PMC5073239

[22] Dierikx CM van Duijkeren E Schoormans AH van Essen-Zandbergen A Veldman K Kant A Occurrence and characteristics of extended-spectrum-β-lactamase- and AmpC-producing clinical isolates derived from companion animals and horses. J Antimicrob Chemother (2012) 67:1368–74. 10.1093/jac/dks049 22382469

[23] Dahmen S Haenni M Châtre P Madec JY. Characterization of *bla*_CTX-M_ IncFII plasmids and clones of *Escherichia coli* from pets in France. J Antimicrob Chemother (2013) 68:2797–801. 10.1093/jac/dkt29 23852541

[24] Huber H Zweifel C Wittenbrink MM Stephan R ESBL-producing uropathogenic *Escherichia coli* isolated from dogs and cats in Switzerland. Vet Microbiol (2013) 162:992–6. 10.1016/j.vetmic.2012.10.029 23177909

[25] Shaheen BW Nayak R Foley SL Kweon O Deck J Park M Molecular characterization of resistance to extended-spectrum cephalosporins in clinical *Escherichia coli* isolates from companion animals in the United States. Antimicrob Agents Chemother (2011) 55:5666–75. 10.1128/AAC.00656-11 21947397 PMC3232758

[26] Zurfluh K Wang J Klumpp J Nüesch-Inderbinen M Fanning S Stephan R Vertical transmission of highly similar *bla*_CTX-M-1_-harboring IncI1 plasmids in *Escherichia coli* with different MLST types in the poultry production pyramid. Front Microbiol (2014) 5:519 10.3389/fmicb.2014.00519 25324838 PMC4179741

[27] Abgottspon H Stephan R Bagutti C Brodmann P Hächler H Zurfluh K. Characteristics of extended-spectrum cephalosporin-resistant *Escherichia coli* isolated from Swiss and imported poultry meat. J Food Prot (2014) 77:112–5. 10.4315/0362-028X.JFP-13-120 24406007

[28] Glaser CA Powers EL Greene CE Zoonotic infections of medical importance in immunocompromised humans. In: Greene CE, editor. Infectious Diseases of the Dog and Cat. St Louis: Elsevier Saunders (2012). p. 1141–62.

[29] Weese JS Rousseau J Arroyo L. Bacteriological evaluation of commercial canine and feline raw diets. Can Vet J (2005) 46:513–6. 16048011 PMC1140397

[30] Baede VO Broens E Spaninks M Timmerman A Graveland H Wagenaar J Raw pet food as a risk factor for shedding of extended-spectrum beta-lactamase producing Enterobacteriaceae in household cats. PLoS One (2017) 12(11):e0187239 10.1371/journal.pone.0187239 29095871 PMC5667807

[31] Rao L Lv L Zeng Z Chen S He D Chen X Increasing prevalence of extended-spectrum cephalosporin-resistant *Escherichia coli* in food animals and the diversity of CTX-M genotypes during 2003-2012. Vet Microbiol (2014) 172:534–41. 10.1016/j.vetmic.2014.06.013 24999233

[32] Woodford N Turton JF Livermore DM. Multiresistant Gram-negative bacteria: the role of high-risk clones in the dissemination of antibiotic resistance. FEMS Microbiol Rev (2011) 35:736–55. 10.1111/j.1574-6976.2011.00268.x 21303394

[33] Wohlwend N Endimiani A Francey T Perreten V. Third-generation-cephalosporin-resistant *Klebsiella pneumoniae* isolates from humans and companion animals in Switzerland: spread of a DHA-producing sequence type 11 clone in a veterinary setting. Antimicrob Agents Chemother (2015) 59:2949–55. 10.1128/AAC.04408-14 25733505 PMC4394820

[34] Ovejero CM Escudero JA Thomas-Lopez D Hoefer A Moyano G Montero N Highly tigecycline-resistant *Klebsiella pneumoniae* sequence type 11 (ST11) and ST147 isolates from companion animals. Antimicrob Agents Chemother (2017) 61(6):e2640–2616. 10.1128/AAC.02640-16 PMC5444156 28396550

[35] Sato T Harada K Usui M Tsuyuki Y Shiraishi T Tamura Y Tigecycline susceptibility of *Klebsiella pneumoniae* complex and *Escherichia coli* isolates from companion animals: the prevalence of tigecycline-nonsusceptible *K. pneumoniae* complex, including internationally expanding human pathogenic lineages. Microb Drug Resist (2017). 10.1089/mdr.2017.0184 29232167

[36] Nicolas-Chanoine MH Bertrand X Madec JY. *Escherichia coli* ST131, an intriguing clonal group. Clin Microbiol Rev (2014) 27:543–74. 10.1128/CMR.00125-13 24982321 PMC4135899

[37] Ghosh H Doijad S Falgenhauer L Fritzenwanker M Imirzalioglu C Chakraborty T *bla*_CTX-M-27_-encoding Escherichia coli sequence type 131 lineage C1-M27 clone in clinical isolates, Germany. Emerg Infect Dis (2017) 23:1754–6. 10.3201/eid2310.170938 28930021 PMC5621564

[38] Birgy A Bidet P Levy C Sobral E Cohen R Bonacorsi S CTX-M-27-producing *Escherichia coli* of sequence type 131 and clade C1-M27, France. Emerg Infect Dis (2017) 23:885 10.3201/eid2305.161865 PMC5403054 28418829

[39] Cambau E Lascols C Sougakoff W Bébéar C Bonnet R Cavallo JD Occurrence of *qnrA*-positive clinical isolates in French teaching hospitals during 2002–2005. Clin Microbiol Infect (2006) 12:1013–20. 10.1111/j.1469-0691.2006.01529.x 16961639

[40] Strahilevitz J Jacoby GA Hooper DC Robicsek A. Plasmid-mediated quinolone resistance: a multifaceted threat. Clin Microbiol Rev (2009) 22:664–89. 10.1128/CMR.00016-09 19822894 PMC2772364

[41] Haenni M Saras E Ponsin C Dahmen S Petitjean M Hocquet D High prevalence of international ESBL CTX-M-15-producing *Enterobacter cloacae* ST114 clone in animals. J Antimicrob Chemother (2016) 71:1497–500. 10.1093/jac/dkw006 26850718

[42] Mora A López C Herrera A Viso S Mamani R Dhabi G Emerging avian pathogenic *Escherichia coli* strains belonging to clonal groups O111:H4-D-ST2085 and O111:H4-D-ST117 with high virulence-gene content and zoonotic potential. Vet Microbiol (2012) 156:347–52. 10.1016/j.vetmic.2011.10.033-22112854

[43] Vangchhia B Abraham S Bell JM Collignon P Gibson JS Ingram PR Phylogenetic diversity, antimicrobial susceptibility and virulence characteristics of phylogroup F *Escherichia coli* in Australia. Microbiology (2016) 162:1904–12. 10.1099/mic.0.000367 27666313

[44] Accogli M Giani T Monaco M Giufrè M García-Fernández A Conte V Emergence of *Escherichia coli* ST131 sub-clone H30 producing VIM-1 and KPC-3 carbapenemases, Italy. J Antimicrob Chemother (2014) 2014(69):2293–6. 10.1093/jac/dku132 24797065

[45] Schaufler K Semmler T Wieler LH Wöhrmann M Baddam R Ahmed N Clonal spread and interspecies transmission of clinically relevant ESBL-producing *Escherichia coli* of ST410 – another successful pandemic clone. FEMS Microbiol Ecol (2016) 92(1):fiv155 10.1093/femsec/fiv155 26656065

[46] Falgenhauer L Imirzalioglu C Ghosh H Gwozdzinski K Schmiedel J Gentil K Circulation of clonal populations of fluoroquinolone-resistant CTX-M-15-producing *Escherichia coli* ST410 in humans and animals in Germany. Int J Antimicrob Agents (2016) 47:457–65. 10.1016/j.ijantimicag.2016.03.019 27208899

[47] Baker KS Dallman TJ Ashton PM Day M Hughes G Crook PD Intercontinental dissemination of azithromycin-resistant shigellosis through sexual transmission: a cross-sectional study. Lancet Infect Dis (2015) 15:913–21. 10.1016/S1473-3099(15)00002-X 25936611

[48] Lin L Nonejuie P Munguia J Hollands A Olson J Dam Q Azithromycin synergizes with cationic antimicrobial peptides to exert bactericidal and therapeutic activity against highly multidrug-resistant gram-negative bacterial pathogens. EBioMedicine (2015) 2:690–8. 10.1016/j.ebiom.2015.05.021 26288841 PMC4534682

[49] Jost C Bidet P Carrère T Mariani-Kurkdjian P Bonacorsi S. Susceptibility of enterohaemorrhagic *Escherichia coli* to azithromycin in France and analysis of resistance mechanisms. J Antimicrob Chemother (2016) 71:1183–7. 10.1093/jac/dkv477 26832755

