# Supplementary material for: High Prevalence of Extended-Spectrum β-Lactamase Producing Enterobacteriaceae Among Clinical Isolates From Cats and Dogs Admitted to a Veterinary Hospital in Switzerland
Source: Front Vet Sci. 2018 Mar 27;5:62. doi: 10.3389/fvets.2018.00062 (PMC5890143; doi:10.3389/fvets.2018.00062)
Supplement: Supplementary file 1 [file table_1.DOCX]

***Supplementary Material***

**High Prevalence of Extended-Spectrum ß-Lactamase producing Enterobacteriaceae among Clinical Isolates from Cats and Dogs admitted to a Veterinary Hospital**

**in Switzerland**

Anna Lena Zogg, Sabrina Simmen, Katrin Zurfluh, Roger Stephan, Sarah Schmitt,

Magdalena Nüesch-Inderbinen*

*** Correspondence**: magdalena.nueesch-inderbinen.uzh.ch

Supplementary Table S1: PCR primers used in this study for targeting virulence and plasmid-mediated antimicrobial resistance genes

| **Primer ID** | **Nucleotide sequence (5'–3')** | **Target** | **Amplicon**  **(bp)** | **Annealing**  **temperature (°C)** | **References** |
| --- | --- | --- | --- | --- | --- |
| **Detection primers for antimicrobial resistance genes** | |  |  |  |  |
|  | |  |  |  |  |
| 2_1_ forward | AAAAATCACTGCGCCAGTTC | *bla*_CTX-M group1_ | 415 | 52 | (Woodford et al., 2006) |
| 3_1_ reverse | AGCTTATTCATCGCCACGTT | *bla*_CTX-M group1_ |  | 52 | (Woodford et al., 2006) |
| 2_2_ forward | CGACGCTACCCCTGCTATT | *bla*_CTX-M group2_ | 552 | 52 | (Woodford et al., 2006) |
| 3_2_ reverse | CCAGCGTCAGATTTTTCAGG | *bla*_CTX-M group2_ |  | 52 | (Woodford et al., 2006) |
| 2_9_ forward | CAAAGAGAGTGCAACGGATG | *bla*_CTX-M group9_ | 209 | 52 | (Woodford et al., 2006) |
| 3_9_ reverse | ATTGGAAAGCGTTCATCACC | *bla*_CTX-M group9_ |  | 52 | (Woodford et al., 2006) |
| Gr. 8 CTX-M-fw | ATG AGA CAT CGC GTT AAG CGG ATG | *bla_CTX-M-_*_group8_ | 829 | 60 | (Zurfluh et al., 2015) |
| Gr. 8 CTX-M-rev | CAC GAC GAC TTT CTG CCT TCT GC | *bla_CTX-M-_*_group8_ |  |  | (Zurfluh et al., 2015) |
| A | CACTCAAGGATGTATTGTG | *bla*_SHV_ | 885 | 50 | (Pitout et al., 1998) |
| B | TTAGCGTTGCCAGTGCTCG | *bla*_SHV_ |  |  | (Pitout et al., 1998) |

Supplementary Table 1 continued

| **Primer ID** | **Nucleotide sequence (5'–3')** | **Target** | | **Amplicon**  **(bp)** | **Annealing**  **temperature (°C)** | **References** |
| --- | --- | --- | --- | --- | --- | --- |
| **Detection primers for antimicrobial resistance genes** | |  |  |  |  |  |
|  |  |  |  |  |  |  |
| aac(6')-Ib_For | TTGCGATGCTCTATGAGTGGCTA | *aac(69)-Ib-cr* | | 482 | 55 | (Park CH, 2006) |
| aac(6')-Ib_Rev | CTCGAATGCCTGGCGTGTTT | *aac(69)-Ib-cr* | |  |  | (Park CH, 2006) |
| C | TCGGGGAAATGTGCGCG | *bla*_TEM_ | | 971 | 50 | (Pitout et al., 1998) |
| D | TGCTTAATCAGTGAGGCACC | *bla*_TEM_ | |  |  | (Pitout et al., 1998) |
| mphAF | GTG AGG AGG AGC TTC GCG AG | *mph(A)* | | 403 | 60 | (Ojo et al., 2004) |
| mphAR | TGC CGC AGG ACT CGG AGG TC | *mph(A)* | |  |  | (Ojo et al., 2004) |
| QEPfor | TGGTCTACGCCATGGACCTCA | *qepA* | | 1137 | 56 | (Karczmarczyk et al., 2010) |
| QEPrev | TGAATTCGGACACCGTCTCCG | *qepA* | |  |  | (Karczmarczyk et al., 2010) |
| QnrAm-F | AGAGGATTTCTCACGCCAGG | *qnrA* | | 516 | 54 | (Cattoir et al., 2007) |
| qnrA_R | GCCATACCTACGGCGATACC | *qnrA* | |  |  | (Robicsek et al., 2006) |
| qnrB_F | GATCGTGAAAGCCAGAAAGG | *qnrB* | | 476 | 54 | (Kim et al., 2009) |
| qnrB_R | ATGAGCAACGATGCCTGGTA | *qnrB* | |  |  | (Kim et al., 2009) |
| qnrC-F | GGGTTGTACATTTATTGAATC | *qnrC* | | 447 | 47 | (Wang et al., 2009) |
| qnrC-R | TCCACTTTACGAGGTTCT | *qnrC* | |  |  | (Wang et al., 2009) |
| qnrD fw | CGAGATCAATTTACGGGGAATA | *qnrD* | | 582 | 54 | (Cavaco et al., 2009) |
| qnrD rev | AACAAGCTGAAGCGCCTG | *qnrD* | |  |  | (Cavaco et al., 2009) |
| QnrSm-F | GCAAGTTCATTGAACAGGGT | *qnrS* | | 428 | 54 | (Cattoir et al., 2007) |
| QnrSm-R | TCTAAACCGTCGAGTTCGGCG | *qnrS* | |  |  | (Cattoir et al., 2007) |
|  |  |  | |  |  |  |
| **Amplifying and sequencing primers** | |  | |  |  |  |
|  |  |  | |  |  |  |
| 1_1_ forward | AAACACACGTGGAATTTAGGG | *bla*_CTX-M group1_ | | 1097 | 52 | (Geser et al., 2012) |
| 4_1_ reverse | CCGTCGGTGACGATTTTAGCC | *bla*_CTX-M group1_ | | 865 |  | (Geser et al., 2012) |
| 5_1_ reverse | CCGATGACTATGCGCACTGGG | *bla*_CTX-M group1_ | |  |  | (Geser et al., 2012) |
| 1_2_ forward | TTTTGCCGTACCTGCGTACCC | *bla*_CTX-M group2_ | | 1187 | 52 | (Geser et al., 2012) |
| 4_2_ reverse | CCGTGGGTTACGATTTTCGCC | *bla*_CTX-M group2_ | | 825 |  | (Geser et al., 2012) |
| 5_2_ reverse | TTGGTCCAGAAAAAAGAGCGG | *bla*_CTX-M group2_ | |  |  | (Geser et al., 2012) |
| 1_9_ forward | TGATGTAACACGGATTGACCG | *bla*_CTX-M group9_ | | 1061 | 52 | (Geser et al., 2012) |
| 4_9_ reverse | AAACCAGTTACAGCCCTTCGG | *bla*_CTX-M group9_ | | 866 |  | (Geser et al., 2012) |
| 5_9_ reverse | TGGAGCCACGGTTGATGAGGG | *bla*_CTX-M group9_ | |  |  | (Geser et al., 2012) |

Supplementary Table 1 continued

| **Primer ID** | | **Nucleotide sequence (5'–3')** | | **Target** | **Amplicon**  **(bp)** | **Annealing**  **temperature (°C)** | **References** | |
| --- | --- | --- | --- | --- | --- | --- | --- | --- |
| **Amplifying and sequencing primers** | | | |  |  |  |  | |
|  | |  | |  |  |  |  | |
| Gr. 8 CTX-M-fw | ATG AGA CAT CGC GTT AAG CGG ATG | | *bla_CTX-M-_*_group8_ | | 829 | 60 | (Zurfluh et al., 2015) | |
| Gr. 8 CTX-M-rev | CAC GAC GAC TTT CTG CCT TCT GC | | *bla_CTX-M-_*_group8_ | |  |  | (Zurfluh et al., 2015) | |
| gyrA WF | | AAATCTGCCCGTGTCGTTGGT | | *gyrA* | 344 | 55 | (Kim et al., 2009) | |
| gyrA WR | | GCCATACCTACGGCGATACC | | *gyrA* |  |  | (Kim et al., 2009) | |
| parC WF | | CTGAATGCCAGCGCCAAATT | | *parC* | 168 | 55 | (Kim et al., 2009) | |
| parC WR | | GCGAACGATTTCGGATCGTC | | *parC* |  |  | (Kim et al., 2009) | |
|  | |  | |  |  |  |  | |
| **Detection primers for virulence genes** | | | |  |  |  |  | |
|  | | | |  |  |  |  | |
| FyuA f | | tgattaaccccgcgacgggaa | | *fyuA* | 880 | 63 | (Johnson and Stell, 2000) |  |
| FyuA r | | cgcagtaggcacgatgttgta | | *fyuA* |  |  | (Johnson and Stell, 2000) |  |
| hly f | | aacaaggataagcactgttctggct | | *hlyA* | 1177 | 63 | (Johnson and Stell, 2000) |  |
| hly r | | accatataagcggtcattcccgtca | | *hlyA* |  |  | (Johnson and Stell, 2000) |  |
| PapA f | | atggcagtggtgtcttttggtg | | *papAH* | 720 |  | (Johnson and Stell, 2000) | |
| PapA r | | cgtcccaccatacgtgctcttc | | *papAH* |  |  | (Johnson and Stell, 2000) | |
| PapEF f | | gcaacagcaacgctggttgcatcat | | *papEF* | 336 |  | (Johnson and Stell, 2000) | |
| PapEF r | | agagagagccactcttatacggaca | | *papEF* |  |  | (Johnson and Stell, 2000) | |
| RPAi f | | ggacatcctgttacagcgcgca | | PAI | 930 | 63 | (Johnson and Stell, 2000) | |
| RPAi r | | tcgccaccaatcacagccgaac | | PAI |  |  | (Johnson and Stell, 2000) | |
| TraT f | | ggtgtggtgcgatgagcacag | | *traT* | 290 | 63 | (Johnson and Stell, 2000) | |
| TraT r | | cacggttcagccatccctgag | | *traT* |  |  | (Johnson and Stell, 2000) | |
| yfcvf | | ACATGGAGACCACGTTCACC | | *yfcv* | 292 | 63 | (Spurbeck et al., 2012) | |
| yfcvr | | GTAATCTGGAATGTGGTCAGG | | *yfcv* |  |  | (Spurbeck et al., 2012) | |

References

Cattoir V, PL, Rotimi V, Soussy C, Nordmann P. (2007). Multiplex PCR for detection of plasmid-mediated quinolone resistance *qnr* genes in ESBLproducing enterobacterial isolates. *J Antimicrob Chemother* 60:394–397.

Cavaco, LM, Hasman, H, Xia, S, Aarestrup, FM. (2009). *qnrD*, a novel gene conferring transferable quinolone resistance in *Salmonella enterica* serovar Kentucky and Bovismorbificans strains of human origin. *Antimicrob Agents Chemother* 53:603–608.

Geser, N., Stephan, R., Korczak, B. M., Beutin, L., Hächler, H. (2012). Molecular identification of extended-spectrum-β-lactamase genes from Enterobacteriaceae isolated from healthy human carriers in Switzerland. *Antimicrob Agents Chemother* 56:1609-1612. doi:10.1128/AAC.05539-11.

Johnson, J. R., Stell, A. L. (2000). Extended virulence genotypes of *Escherichia coli* strains from patients with urosepsis in relation to phylogeny and host compromise. *J Infect Dis*, 181, 261-272. doi:10.1086/315217.

Karczmarczyk, M, Martins, M, McCusker, M, Mattar, S, Amaral, L, Leonard, N, Aarestrup, FM, Fanning, S. (2010). Characterization of antimicrobial resistance in *Salmonella enterica* food and animal isolates from Colombia: identification of a *qnrB19*-mediated quinolone resistance marker in two novel serovars. *FEMS Microbiol Lett* 313:10–19.

Kim, HB, Park, CH, Kim, CJ, Kim, E-C, Jacoby, GA, Hooper, DC. (2009). Prevalence of plasmid-mediated quinolone resistance determinants over a 9-year period. *Antimicrob Agents Chemother* 53:639–645.

Ojo, K. K., Ulep, C., Van Kirk, N., Luis, H., Bernardo, M., Leitao, J., and Roberts, M. C. (2004). The *mef(A*) gene predominates among seven macrolide resistance genes identified in gram-negative strains representing 13 genera, isolated from healthy Portuguese children. *Antimicrob Agents Chemother* 48: 3451-3456. doi:10.1128/AAC.48.9.3451-3456.2004.

Pitout, JD, Thomson, KS, Hanson, ND, Ehrhardt, AF, Moland, ES, Sanders, CC. (1998). beta-lactamases responsible for resistance to expanded-spectrum cephalosporins in *Klebsiella pneumoniae*, *Escherichia coli,* and *Proteus mirabilis* isolates recovered in South Africa. *Antimicrob Agents Chemother* 42:1350–1354.

Robicsek, A, Strahilevitz, J, Sahm, DF, Jacoby, GA, Hooper, DC. (2006). *qnr* prevalence in ceftazidime-resistant Enterobacteriaceae isolates from the United States. *Antimicrob Agents Chemother* 50:2872–2874.

Spurbeck, RR, Dinh, PC, Walk, ST, Stapleton, AE, Hooton, TM, Nolan, LK, Kim, KS, Johnson, JR, Mobley, HLT. (2012). *Escherichia coli* isolates that carry *vat*, *fyuA*, *chuA*, and *yfcV* efficiently colonize the urinary tract. *Infect Immun* 80:4115–4122.

Wang, M, Guo, Q, Xu, X, Wang, X, Ye, X, Wu, S, Hooper, DC, Wang, M. (2009). New plasmid-mediated quinolone resistance gene, *qnrC*, found in a clinical isolate of *Proteus mirabilis*. *Antimicrob Agents Chemother* 53:1892–1897.

Woodford, N., Fagan, E. J., and Ellington, M. J. (2006). Multiplex PCR for rapid detection of genes encoding CTX-M extended-spectrum β-lactamases. *J. Antimicrobl Chemother.* 57, 154-155.

Zurfluh, K., Nüesch-Inderbinen, M., Morach, M., Berner, A. Z., Hächler, H., Stephan, R. (2015). Extended-spectrum ß-lactamase-producing-Enterobacteriaceae in vegetables imported from the Dominican Republic, India, Thailand and Vietnam. *Appl Environ Microbiol*, 81, 3115-3120. doi:10.1128/AEM.00258-15.
