# Supplementary material for: High Prevalence of Extended-Spectrum β-Lactamase Producing Enterobacteriaceae Among Clinical Isolates From Cats and Dogs Admitted to a Veterinary Hospital in Switzerland
Source: Front Vet Sci. 2018 Mar 27;5:62. doi: 10.3389/fvets.2018.00062 (PMC5890143; doi:10.3389/fvets.2018.00062)
Supplement: Supplementary file 3 [file table_3.DOCX]

***Supplementary Material***

**High Prevalence of Extended-Spectrum ß-Lactamase producing Enterobacteriaceae among Clinical Isolates from Cats and Dogs admitted to a Veterinary Hospital in Switzerland**

Anna Lena Zogg, Sabrina Simmen, Katrin Zurfluh, Roger Stephan, Sarah N Schmitt,

Magdalena Nüesch-Inderbinen*

*** Correspondence**: magdalena.nueesch-inderbinen@uzh.ch

^*^

Table 3. Results of the MLST analysis for *E. coli* strains with new STs.^a^

| **Isolate ID** | **PG** |  | **MLST alleles** | | | | | | |  | **ST** | **Related ST(CC)** |
| --- | --- | --- | --- | --- | --- | --- | --- | --- | --- | --- | --- | --- |
|  |  |  | *adk* | *fumC* | *gyrB* | *icd* | *mdh* | *purA* | *recA* |  |  |  |
| 3498 | D |  | 154 | 187 | 22 | 1 | 130 | 129 | 4 |  | New | None (none) |
| 2943 | A |  | 10 | 99 | 5 | 91 | 273 | 7 | 2 |  | New | ST361 (none |
| 2236 | C |  | 6 | 4 | 193 | 1 | 20 | 18 | 7 |  | New | ST410 (CC23) |

^a^The ST were not assigned numerical designations by the *E. coli* MLST database (http://mlst.warwick.ac.uk/mlst/dbs/Ecoli).

CC, clonal complex; MLST, multilocus sequence type; PG, phylogenetic group; ST, sequence type.
